# Therapeutic approach to pediatric acute mastoiditis – an update^☆^

**DOI:** 10.1016/j.bjorl.2018.06.002

**Published:** 2018-07-17

**Authors:** Józef Mierzwiński, Justyna Tyra, Karolina Haber, Maria Drela, Dariusz Paczkowski, Michael David Puricelli, Anna Sinkiewicz

**Affiliations:** a Children's Hospital of Bydgoszcz, Pediatric Cochlear Implant Center, Department of Otolaryngology, Audiology and Phoniatrics, Bydgoszcz, Poland; b Paparella Ear, Head & Neck Institute, Minneapolis, United States; c Nicolaus Copernicus University Hospital of Bydgoszcz, Department of Health Sciences, Department of Phoniatrics and Audiology, Bydgoszcz, Poland

**Keywords:** Mastoiditis, Child, Recurrence, Mastoidectomy, Cochlear implants, Mastoidite, Criança, Recorrência, Mastoidectomia, Implantes cocleares

## Abstract

**Introduction:**

Acute mastoiditis remains the most common complication of acute otitis media. It may rarely appear also in cochlear implant patients. However, the treatment recommendations for this disease are not precisely defined or employed, and in the current literature the differences regarding both the diagnosis and management are relatively substantial.

**Objective:**

The aim of this study was to determine a standard and safe procedure to be applied in case of pediatric acute mastoiditis.

**Methods:**

A retrospective chart review of 73 patients with 83 episodes of acute mastoiditis hospitalized at our tertiary-care center between 2001 and 2016 was conducted. Bacteriology, methods of treatment, hospital course, complications, and otologic history were analyzed. Based on our experience and literature data, a protocol was established in order to standardize management of pediatric acute mastoiditis.

**Results:**

All the patients treated for acute mastoiditis were submitted to an intravenous antibiotic regimen. In the analyzed group pharmacological treatment only was applied in 11% of children, in 12% myringotomy/tympanostomy was added, and in the vast majority of patients (77%) mastoidectomy was performed. In our study recurrent mastoiditis was noted in 8% of the patients. We also experienced acute mastoiditis in a cochlear implant child, and in this case, a minimal surgical procedure, in order to protect the device, was recommended.

**Conclusions:**

The main points of the management protocol are: initiate a broad-spectrum intravenous antibiotic treatment; mastoidectomy should be performed if the infection fails to be controlled after 48 h of administering intravenous antibiotic therapy. We believe that early mastoidectomy prevents serious complications, and our initial observation is that by performing broad mastoidectomy with posterior attic and facial recess exposure, recurrence of acute mastoiditis can be prevented.

## Resumo

**Introdução:**

A mastoidite aguda continua a ser a complicação mais comum da otite média aguda. Pode ocorrer também, embora raramente, em pacientes com implante coclear. Entretanto, as recomendações de tratamento para essa doença não são bem definidas ou usadas e, na literatura corrente, as diferenças em relação ao diagnóstico e ao manejo são relativamente significativas.

**Objetivo:**

O objetivo deste estudo foi determinar um procedimento padrão e seguro a ser aplicado em caso de mastoidite aguda pediátrica.

**Método:**

Foi realizada uma revisão retrospectiva de prontuários de 73 pacientes com 83 episódios de mastoidite aguda hospitalizados em nosso centro terciário entre os anos de 2001 a 2016. Foram analisados a bacteriologia, métodos de tratamento, evolução hospitalar, complicações e histórico otológico. Com base em nossa experiência e dados da literatura, foi estabelecido um protocolo para padronizar o tratamento da mastoidite aguda pediátrica.

**Resultados:**

Todos os pacientes tratados para mastoidite aguda foram submetidos a antibioticoterapia endovenosa. No grupo analisado, o tratamento farmacológico só foi aplicado em 11% das crianças, em 12% a miringotomia/timpanostomia foi adicionada e na maior parte dos pacientes (77%) foi feita a mastoidectomia. Em nosso estudo, mastoidite recorrente foi observada em 8% dos pacientes. Também observamos mastoidite aguda em criança usuária de implante coclear e, nesse caso, foi recomendada a minimização de procedimentos cirúrgicos, a fim de proteger o dispositivo.

**Conclusões:**

Os principais pontos do protocolo de conduta são: iniciar um tratamento antibiótico endovenoso de amplo espectro; a mastoidectomia deve ser feita caso a infecção não seja controlada após 48 horas da administração de antibioticoterapia intravenosa. Acreditamos que a mastoidectomia precoce previne complicações graves e nossa observação inicial é que, com uma mastoidectomia ampla com exposição do ático posterior e do recesso facial, a recorrência de mastoidite aguda pode ser evitada.

## Introduction

Acute otitis media (AOM) is one of the most frequent diseases in young children, especially in children aged 6–24 months.^1^ Hassman et al., reported that 80% of children have suffered at least one episode of AOM by their third birthday.^2^ The most common complication of AOM is acute mastoiditis (AM). AM is a middle ear infection that extends to the mastoid cells and involves the mastoid bone, leading to periostitis and/or osteitis.^3^ An inflammatory process in the mastoid air cell system occurs in any case of acute otitis media, however not every case leads to AM. In the case of AM, the direct cause of retention and increase of pus pressure in the mastoid process is the loss of communication between the mastoid cavity and tympanic cavity, preventing drainage through the eustachian tube or tympanic membrane (TM) perforation.

Usually the loss of communication is caused by the blockage, which occurs in the aditus ad antrum and can be caused by the inflammation of the mucosal lining in the middle ear. Inflammatory fluid accumulation and pressure increase of purulent secretions may lead to osteolysis of mastoid cells and predispose to formation of abscesses and pathological fistulas.^4^, ^5^ This kind of inflammation with the retention of secretion in mastoid cells, inability to drain, and osteolysis is defined as AM.

Restoring communication between the tympanic cavity and the mastoid cavity with intensive drug treatment – parenteral antibiotic therapy, or if it is not effective through surgical treatment – drainage of the subperiosteal abscess or antromastoidectomy, is a fundamental form of treating pediatric patients with mastoiditis. An additional advantageous option of correcting the drainage of the middle ear is incision of the tympanic membrane and insertion of ventilation tubes.^6^ AM appears with incidence 0.6–4.2/100,000 children per year.^4^, ^7^ Epidemiological differences are frequently due to standards of acute otitis media treatment in various regions of the world. As it is widely known that the majority of AOM cases can be successfully treated without antibiotics, so the algorithms of management vary from country to country. In the countries where recommendations suggest the use of antibiotics in almost every single AOM case, epidemiology of AM seems to be lower than in countries where the use of antibiotics is restricted to small children and more severe cases only.^7^ As for the age distribution, children are more prone to AM, since pneumatization of the mastoid process begins just after birth and is completed when the child is approx. 10 years old. Luntz et al., reported that in the group of 223 children with AM, 28% of the children were under the age of 1, 38% were aged 1–4 years, 21% 4–8 and 12% were older, up to 18 years old. 30% of children suffered from recurrent otitis media episodes before AM.^8^

In spite of modern standards in the treatment of AOM and wide use of antibiotics in the treatment of this disease AM is still relatively frequently encountered, and in some cases, can lead to intracranial or intratemporal complications.^9^

Infrequently observed problems are also recurrent cases of AM, which according to our own experience, accounts for 2–18% of the cases.^8^, ^10^, ^11^, ^12^, ^13^ Very little literature data is available on the subject.

Current recommendations on the management of AOM are precisely defined and implemented in most countries, according to national programs.^14^ However, the recommendations for AM are not so clearly defined or used. There are also new, atypical cases – entities like AM after cochlear implantation (CI) which should also be interpreted, and to some extent standardized. In the current literature the differences regarding both the diagnosis and management of AM in children are relatively substantial.^3^, ^4^, ^8^, ^15^, ^16^, ^17^, ^18^

The aim of this study was to determine a standard and safe procedure to be applied in cases of AM in children, based on a retrospective analysis of a group of children admitted and treated in our center.

## Methods

The study was completed in accordance with all procedures performed in studies involving human participants and in accordance with the ethical standards of the institutional and/or national research committee and with the 1964 Helsinki declaration and its later amendments or comparable ethical standards. Bioethical committee permission number: KB 582/2017.

A retrospective study was conducted on children diagnosed with AM and treated between 2001 and 2016 in our department. The second or third episode of acute mastoiditis was noted as recurrent. Recurrence was defined as a new episode of AM requiring hospitalization with all clinical symptoms typical for acute mastoiditis. If the second episode occurred within 4 weeks from the onset of the disease – it was qualified as the same episode, and was not classified as a recurrent acute mastoiditis (rAM). Two children who were treated for AM in the selected period were excluded from further analysis because of AM, due to the oncological process and temporal bone fibrous dysplasia with secondary atresia of auditory meatus and cholesteatoma. Additionally, the children who had AM as the result of chronic otitis media or cholesteatoma, were also excluded. Diagnosis and classification of the treatment was based on both subjective and objective examinations (ear pain, hearing loss, protrusion of the ear, post auricular swelling, occlusion and lowering of the posterior-upper wall of the ear canal). In cases where the diagnosis was unclear, the course of disease unfavorable, or suspicion of intracranial or intratemporal complications occurred, children were referred for diagnostic imaging, including CT of the temporal bone and alternatively MRI with contrast.

On admission we used sterile swab-sticks to collect a specimen of the ear discharge. In cases of surgical intervention, the pus was collected during surgery.

**Figure 1:**
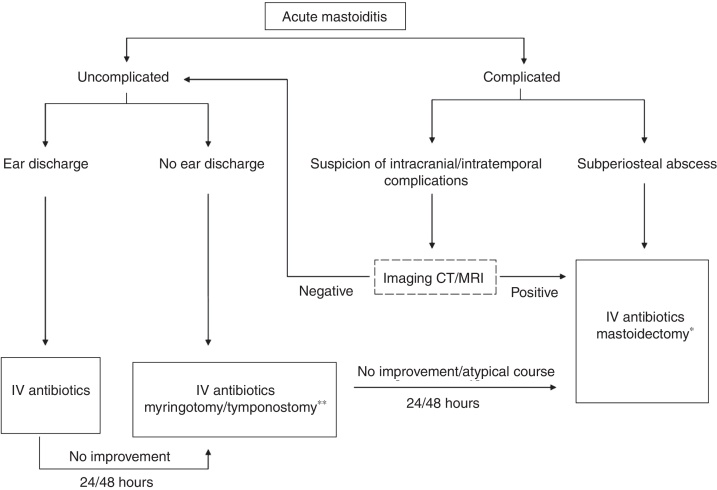
Management algorithm of acute mastoiditis in children. *At present in acute mastoiditis we recommend mastoidectomy with wide exposure of attic and posterior tympanotomy in order to provide broad communication between tympanic and mastoid cavity. We do not recommend incision and drainage of subperiosteal abscess but mastoidectomy in these cases. One exception is post cochlear implant mastoiditis were such conservative abscess drainage treatment is accepted and recommended due to electrodes and CI body in the operative field. As a rule tympanostomy is performed during the procedure. ** If no or scant ear discharge – to create adequate drainage we always try to insert ventilating tubes (tympanostomy), but in case of thick inflammatory tympanic membrane it is sometimes difficult and in this situation myringotomy is performed only.

Management of AM included administering empiric intravenous antibiotics shortly after the admission in every single case (Fig. 1). In case of severe ear pain and elevation of the TM without perforation, the treatment was supplemented with paracentesis and insertion of a tympanostomy tube into the eardrum. If after 24–48 h no evident clinical improvement was achieved, children were recommended for a surgical treatment, either myringotomy, tympanostomy tube insertion or mastoidectomy.

The questionnaire regarding previous episodes of acute otitis media had been sent to all the patients treated due to AM.

Categorical data were statistically analyzed using a Chi-Square Test and Fisher's exact test.

## Results

From 2001 to 2016, 83 ears diagnosed with AM in 73 patients were treated in our department. There were 35 boys and 38 girls. The patient's age varied from 4 months to 18 years. The largest group (51%) of AM cases comprised of children aged 1–4 years (Table 1).

**Table 1:** Age distribution in 73 patients with acute mastoiditis.

Age (years)	Number of patients (%)
<1	8 (11%)
1–4	37 (51%)
4–8	18 (24%)
>8	10 (14%)

Mean hospitalization time was 11.34 days. The second or third episode of AM was noted in 6 patients. In 4 children AM was bilateral.

**Figure 2:**
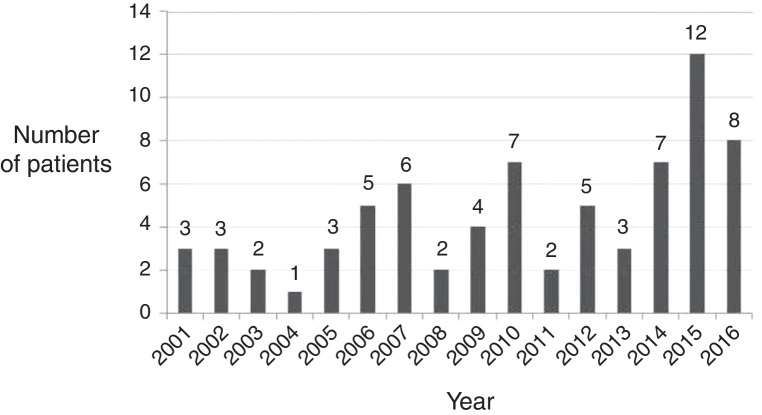
Cases of acute mastoiditis treated in our department in years 2001–2016, significant increase.

We are a major tertiary referral center in the region meeting the needs of about 2 million inhabitants, thus, having collected the data concerning the children in our area (varying between 2001 and 2016 from approx. 370,000 to 500,000 children) and having established the number of children diagnosed with AM (obtained from the annual statistics for H70 ICD-10 coding), we have determined the epidemiology of AM. The counted incidence rate is 1.86/100,000 per year, considering children 0–18 years old. We have observed statistically significant (*p* < 0.05; the Chi-Square Test was used) increase in the rate of incidence during the last years (Table 2, Fig. 2).

**Table 2:** Incidence of acute mastoiditis in Kuyavian-Pomeranian Region of Poland between the years 2001–2015. We have observed statistically significant (*p* < 0.05; the Chi-Square Test was used) increase in the rate of incidence during the last years.

Year	Number of children in the region	Hospital – number of children with AM	Region – number of children with AM	Incidence per 100,000 children	Average annual incidence in the region
2015	378,825	12	15	3.959612	1.863559223 ≈ 1.86
2014	383,177	7	8	2.087808	
2013	387,933	3	6	1.546659	
2012	393,346	5	10	2.542291	
2011	399,592	2	9	2.252297	
2010	406,742	7	10	2.458561	
2009	406,397	4	5	1.230324	
2008	413,722	2	5	1.208541	
2007	421,767	6	7	1.659684	
2006	431,113	5	7	1.623704	
2005	441,718	3	5	1.131944	
2004	453,662	1	3	0.661285	
2003	467,670	2	No data	No data	
2002	484,870	3	No data	No data	
2001	503,071	3	No data	No data

We are practically the only institution treating AM in the area and only single cases of mastoiditis, which resolve on conservative treatment, are treated outside our institution. The dominant pathogen in our group was Streptococcus pneumoniae – 33.7% of cases (Table 3).

**Table 3:** Microbiological examination results.

Isolated pathogen	Number of ears (*n* = 83)	(%)
*Streptococcus pneumoniae*	28	33.7%
*Streptococcus pyogenes*	13	15.7%
*Pseudomonas aeruginosa*	3	3.6%
*Haemophilus influenzae*	3	3.6%
*Candida albicans*	2	2.4%
*Escherichia coli*	1	1.2%
*Acinetobacter baumannii*	1	1.2%
*Enterococcus faecalis*	1	1.2%
*Staphylococcus aureus*	1	1.2%
*Moraxella catarrhalis*	1	1.2%
**Negative**	29	35%

All the patients treated for AM or rAM were submitted for intravenous antibiotic regimen. According to the bacteriological status, blood test results and clinical course of the disease, patients relied on antibiotics only or were recommended for further therapy.

As a first-line antibiotic treatment, we usually used the combination of the 3rd generation cephalosporin (ceftriaxone or cefotaxim) with clindamycin or metronidazole. Such combination has covered the bacteria most usually involved in AM, and has proven to be effective in most cases. Antibiotics were secondarily adapted to the results of the microbiological samples if necessary.

The mode of treatment from conservative through myringotomy/tympanostomy to mastoidectomy in the whole group of acute mastoiditis is given in Table 4.

**Table 4:** Management of children treated due to acute mastoiditis (83 ears).

Mastoidectomy only	Mastoidectomy + tympanostomy	Tympanostomy/myringotomy	Pharmacological treatment only
8 (10%)	56 (67%)	10 (12%)	9 (11%)

In case of profuse ear discharge we did not use myringotomy/tympanostomy as an initial treatment. We did not use incision and drainage of patients with subperiosteal abscess, since these patients had been qualified to urgent mastoidectomy.

Posterior tympanotomy was performed in 15.66% (13 ears). It is very important to note that none of the patients after initially-performed mastoidectomy with wide attic exposure and posterior tympanotomy developed the second episode of AM. A relatively small number of patients were not sufficient to perform relevant statistical analysis.

Recurrent mastoiditis appeared in 6 (8%) of patients. Whereas the sex of the child was insignificant in the first episode of AM (*p* < 0.05; the Chi-Square Test was used), in the recurrent cases we found predominantly boys (five boys and one girl) yet the sample size was too small to perform statistical analysis. The time of the subsequent episode of AM from the first episode varied from 2 months to 3 years. 4 out of 6 patients with recurrent episode of AM underwent surgery twice (mastoidectomy and remastoidectomy). The second/third procedure involved wide mastoidectomy, and in order to obtain broad communication between tympanic and mastoid cavity, the aditus ad antrum was cleaned and exposed. Additionally, posterior tympanotomy was performed which enabled an additional drainage pathway. In one patient, initially treated surgically with mastoidectomy, the second episode was treated pharmacologically. In another patient previously treated pharmacologically the second episode was subjected to mastoidectomy. The decision for further treatment was related to the clinical course of the disease, additional complications, blood test results and, if performed, the results of the imaging (undertaken on the case by case basis). In 5 out of 6 patients with the second episode of mastoiditis the history of recurrent otitis media was noted and the result was statistically significant (*p* < 0.05) compared to the whole group of AM patients (Fisher's exact test was used for statistical analysis). In one rAM child the first episode of AM was the first manifestation of otologic disease (Table 5).

**Table 5:** History of acute otitis media in 73 AM patients.

	Yes	No	No data – 38 patients
Earlier single episodes of otitis media	20/35 (57%)	15/35 (43%)	38/73 (52%)
Recurrent otitis media (4 or more episodes of AOM within the previous 12 months).	12/35 (34%)	23/35 (66%)	

One patient had 3 episodes of AM, so the third time during the mastoidectomy the obliterated aditus ad antrum was opened widely, posterior tympanotomy performed and silastic inserted in order to keep drainage in the area. One patient with rAM was a cochlear implant patient with pathology at implant site. The most frequent complication was sigmoid sinus thrombophlebitis. The list of intratemporal/intracranial complications encountered is shown in Table 6.

**Table 6:** Intracranial/intratemporal complications.

Complication	*n* = 83
Epidural abscess	1 patient (1.2%)
Sigmoid sinus thrombophlebitis	4 patients (5%)
Petrositis	1 patient (1.2%)
Facial paralysis	1 patient (1.2%)

A subperiosteal abscess was observed in 19.3% (16 cases). Imaging studies MRI/CT were performed in patients with persistent disease or if intracranial/intratemporal complications had been suspected. All the complications were diagnosed and revealed in the patients at the time of, or shortly after admission when imaging studies were carried out. No intracranial/intratemporal complication occurred during the treatment and all the patients fully recovered from their disease. Children admitted with the suspected complication were submitted to imaging studies and mastoidectomy immediately.

The questionnaire results confirm the observation that in many cases of AM it is the first manifestation of otologic disease in a child (Table 5). 43% of the mothers did not remember any episode of acute otitis media before the AM episode. 57% children had a single episode of AOM, 34% suffered from recurrent otitis media, and 8% of children had prior episode of AM.

## Discussion

The significant increase in the AM incidence during recent years (Fig. 2) noted in this study and the study of Van Zuijlen can be ascribed to pediatric recommendations for limited use of antibiotics in the treatment of acute otitis media.^7^ Historically, the standard treatment of AM used to be drainage of mastoid cavity by simple mastoidectomy. Introduction of broad spectrum antibiotics decreased the incidence of AM in general, and enabled pharmacological treatment in less advanced cases of mastoiditis.^19^

At present there are many reports supporting a conservative approach to the treatment of AM. Some authors discourage performance of mastoidectomy, considering the risk of mastoidectomy complications itself.^16^, ^20^ Some authors also are against performing even minor surgical procedures, i.e. myringotomy/tympanostomy or incision and drainage of subperiosteal pocket, relying exclusively on the antibiotic therapy as standard treatment.^20^ However, it should be carefully determined whether prolonged conservative treatment is always safe and will not lead to intracranial/intratemporal complications.

Pedersen et al., reported a group of 79 patients with AM.^18^ In all cases mastoidectomy was performed and turned out to be a very effective treatment with no longterm negative impact on the child, minimal rate of complications, thus being reported as a treatment of choice in acute AM.^18^

Lahav performed mastoidectomy only in 11% of 78 children with AM, in other patients with subperiosteal abscess used single or multiple abscess aspiration.^16^ One child developed epidural abscess after such treatment. Vassbotn et al., recommend vigilance when treating AM conservatively, and report a case treated for one week with antibiotics who developed meningitis and epidural abscess.^4^ According to Pang et al., among 79 patients 46% were treated conservatively without any surgery, but the percentage of dangerous complications was higher than in our group, treated more aggressively with mastoidectomy in majority of cases.^21^ The main question that should be addressed is where is the borderline between conservative and surgical management – how much conservative treatment is safe?^22^

Having reviewed literature data, it can be stated that the differences between the centers regarding performing mastoidectomy are wide and vary from 9% to 88%.^4^, ^16^, ^18^, ^21^, ^22^, ^23^ On the basis of 73 children treated in our center we can conclude that AM treatment protocol suggested proved to be safe and helped us to successfully cure the patients with AM (Fig. 1).

The treatment of the first/second/third episode of AM can be:

• Only pharmacological;
• Pharmacological with myringotomy/tympanostomy tube insertion;
• Pharmacological with incision and drainage of subperiosteal abscess;
• Pharmacological with mastoidectomy/re-mastoidectomy.

Obviously, each patient has to be evaluated on the individual, case by case basis. However, the elaboration of a safe algorithm of AM treatment is very helpful for young doctors on duty in hospitals where children with AM may be encountered.

The discussion as to the treatment pertains mainly to the decision about which patient and for how long each can be safely treated only pharmacologically, when to add myringotomy/tympanostomy, and when to perform abscess drainage/or mastoidectomy. Should simple mastoidectomy or combined surgery to create broad communication and drainage to the tympanic cavity (posterior attic exposure, posterior tympanotomy) be chosen.

There is general consensus that all the AM patients should get intravenous antibiotic treatment. Empirically the choice should be the 3rd generation cephalosporin (ceftriaxone or cefotaxim), frequently combined with clindamycin or metronidazole. All authors agree also that subperiosteal abscess should be resolved surgically, and the options are: repeated aspiration/incision and drainage of abscess or mastoidectomy.

Repeated aspiration in children may be difficult because of poor cooperation, and may thus require repeated anesthesia, so we do not recommend it. Incision and drainage of a periauricular abscess is a relatively good option but also due to frequent poor cooperation, daily wound toilet is somewhat difficult in children. In some cases it also requires multiple episodes of general anesthesia. Aspiration/incision also does not secure constant drainage of mastoid cavity, which can cause repeated accumulation of pus in the middle ear spaces. Another relative disadvantage of aspiration is the inability to expose other existing complications of acute mastoiditis, such as epidural abscess or sinus vein thrombosis, which can be detected and immediately treated during mastoidectomy. Therefore, we do recommend mastoidectomy also in these situations.

In the analyzed group, pharmacological treatment only was used in 11% of children, in 12% myringotomy/tympanostomy was added, and in the vast majority of patients – 77%-mastoidectomy was performed as the basic treatment with or without myringotomy/grommet insertion (Table 4). We usually left the drain tube behind the ear for 7 days after the surgery to enable additional drainage of mastoid secretions. It has to be emphasized that all the children were cured and in none of the patients intracranial/intratemporal complications developed during hospitalization or even long thereafter. The recurrent cases also had no serious complication. In 8% (6 patients) the second/third episode of AM was noted and 5 of them were treated surgically again. In all of these cases wide revision of mastoid cavity with the removal of granulous tissue and bone around the “aditus ad antrum” was applied. Additionally, to enable broad communication between mastoid and tympanic cavity posterior tympanotomy was performed. No post-mastoidectomy complications were observed. Each of our patients was submitted to intravenous antibiotic therapy, and in case of profuse ear discharge myringotomy/tympanostomy was considered unnecessary. In cases of otalgia and no drainage, myringotomy/tympanostomy in general anesthesia was performed. It should be noted that in every case of myringotomy inserting grommets is preferable. However, in some cases, where a very narrow ear canal and thick tympanic membrane makes this procedure extremely difficult, only myringotomy was done. If no clinical improvement after 24–48 h was observed, the children were referred for mastoidectomy. Patients with subperiosteal abscess and possible intracranial/intratemporal complications were referred for urgent mastoidectomy.

The question to discuss is the necessity of perform imaging studies in children with acute mastoiditis. Some authors suggest that CT should be a standard procedure in the diagnosis of every AM case.^24^, ^25^ The majority of authors however, support the opinion that imaging studies should be limited to selected cases of AM, where an atypical course of the disease is observed, or where an intracranial/intratemporal complication is suspected.^3^, ^16^, ^22^, ^26^ Also the recurrent cases should undergo imaging studies. The group which was also referred for the CT scan were patients where otoscopy was impossible due to diffuse edema (in order to differentiate from external otitis).

Recurrence rate was 8% of all the treated AM cases.

It is well known that lack of communication with subsequent accumulation of pus in mastoid space is the main cause of AM. Theoretically, it should encourage the surgeon to apply broad mastoidectomy and perform a wide communication path of drainage (attic cleaning and facial recess exposure) in every single patient with AM, where mastoidectomy is required. It is highly probable that such a treatment may prevent secondary AM. Based on these assumptions we started to introduce such a treatment and performed it in 13 cases. In primary AM cases where such a treatment has been implemented not a single case of rAM has been observed until now.^27^ We also opened the facial recess in all cases of recurrent disease. Even in the cases where granulation tissue, or bone regrowth may secondarily lead to lack of communication and cause mastoiditis in the presence of infection, we believe that in these cases the recurrence in less likely. Similar experience was reported by Lindner et al., as their recurrent AM patients previously treated with simple AM developed rAM.^17^ At the second time remastoidectomy with wide exposure of facial recess was performed.^17^ Linder's paper is the only article where the problem is mentioned pertaining to AM. The concept of adding posterior tympanotomy to simple mastoidectomy in AM cases to improve middle ear communication had also been previously suggested by Sade.^28^ At present, after the initial observations, we have adopted it as a rule that in every case of acute mastoiditis, where indications for mastoidectomy are established, we perform attic exposure with clearance, and posterior tympanotomy. Future observations will confirm our initial presumptions, and confirm whether the numbers of recurrence will be lower than in children after simple mastoidectomy, or conservative treatment.

There are also relatively new situations where we also decided to adopt some algorithm of treatment as for AM. These concern the AM patients after cochlear implantations. Cochlear implants are used in very small children who are vulnerable to acute otitis media infection and mastoiditis infections.^29^ Rodriguez et al., reported the incidence of otitis media in 31–61% of CI children prior to CI and with 28% incidence of post CI surgery.^30^ According to their research it was the result of natural growth process and children's immune system development, not mastoidectomy applied as preventive measures for recurrence of the AOM disease. Serious middle ear infections including AM are estimated for 1–2% of CI users.^30^, ^31^ It should be emphasized that in CI users each middle ear infection can spread along the electrodes to the inner ear and central nervous system causing subsequent intracranial complications. The management here is of special interest and in worst cases may end up in explantation of the CI which means, at least temporarily, loss of hearing for the patient. We do recommend in this group of patients more conservative treatment for AM. Intravenous antibiotics application remains the basis of the treatment, and if surgical drainage is necessary abscess incision and drainage are recommended. Mastoidectomy in the presence of wires and CI electrodes is really stressful for the surgeon as it makes manipulations very difficult. In our CI patient AM developed 2 years after the surgery. We tried antibiotic treatment with grommet insertion but without initial improvement. Due to the lack of improvement we decided to incise the abscess, insert the drain and rinse the mastoid cavity with antibiotics once daily for a week, with good effect. Explantation is only seldom necessary in these cases.^31^, ^32^ Theoretically, the indication for explantation would be wide skin fistula exposing the implant site, labyrinthitis with subsequent meningitis, complete lack of therapeutic improvement, or implant failure.^27^ Migirov described 11 patients with AM, which appeared after the CI.^12^ In 3 cases he incised the subperiosteal abscess, while in 9 pharmacological treatment turned out to be effective. No implant removal was necessary in his studies. Zawawi, analyzed 43 CI users with AM, and in his research explantation was necessary in only one patient (2.3%), mainly due to ineffective pharmacological and surgical treatment and severe pain accompanied by cochlear stimulation.^31^ After the explanation all the symptoms disappeared. It has to be mentioned that the second episodes of AM also appear in children with cochlear implants.^31^

In our study it has been proven that both in AM and recurrent AM cases Streptococcus pneumoniae is the main pathogen responsible for mastoiditis and recurrences. Similar observations and rate were also reported by other authors.^4^, ^8^, ^10^, ^18^, ^21^

Since January 1, 2017 the vaccination against Streptococcus pneumoniae has been obligatory in Poland. It will be interesting to see whether Pneumococcal vaccination will decrease the incidence of AM and rAM, and if mastoidectomy extended to facial recess will prevent rAM episodes in the future.

## Conclusions

In conclusion acute mastoiditis can be safely treated with the following therapeutic guidelines proposed to standardize the management of AM.

The treatment should be started with obtaining bacteriological specimen and empiric intravenous antibiotic treatment effective for Streptococcus pneumoniae and additional bacteria most frequently encountered. In cases were no ear discharge is present myringotomy/tympanostomy should be contemplated. If no improvement is observed within 24–48 h following the initial treatment, or if any complications are suspected, extension to surgical procedures should be considered: mastoidectomy (with the option of attic exposure and facial recess opening), or in case of cochlear implant ear AM – abscess incision and drainage is recommended. Looking at our group we can assume that early surgical intervention in the treatment of AM prevents development of serious intracranial/intracochlear complications.

## Conflicts of interest

The authors declare no conflicts of interest.
